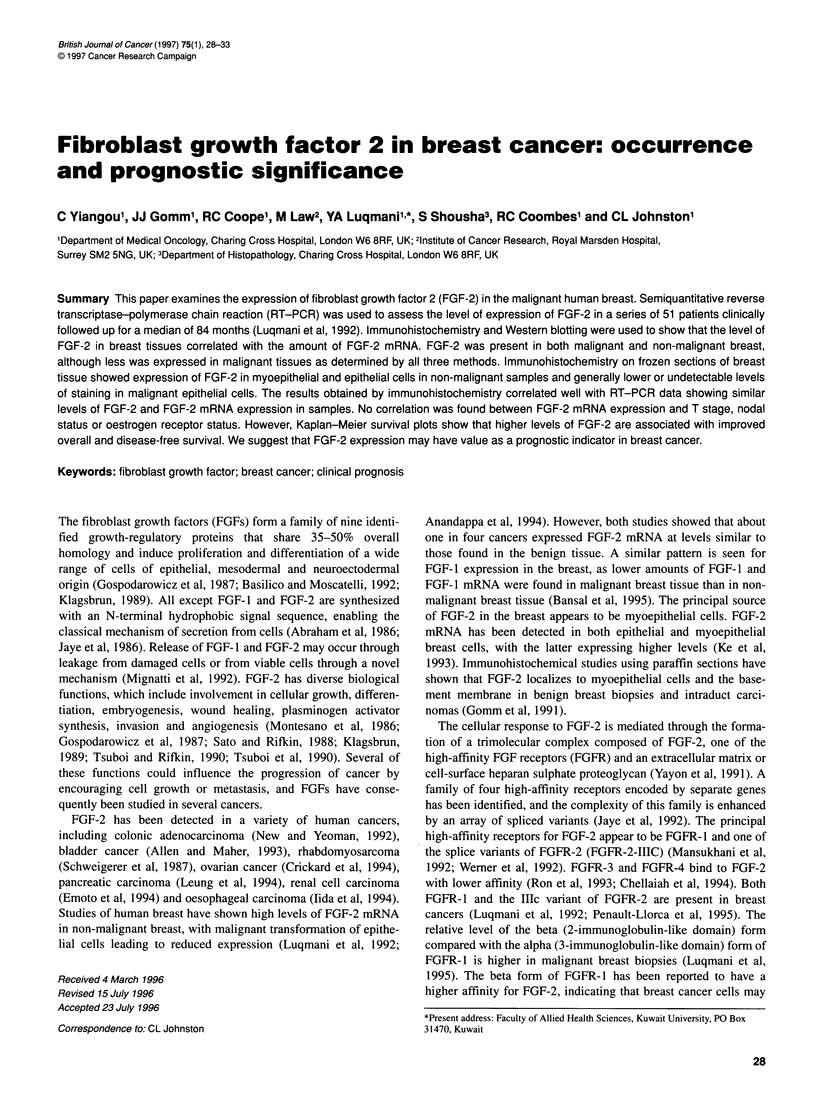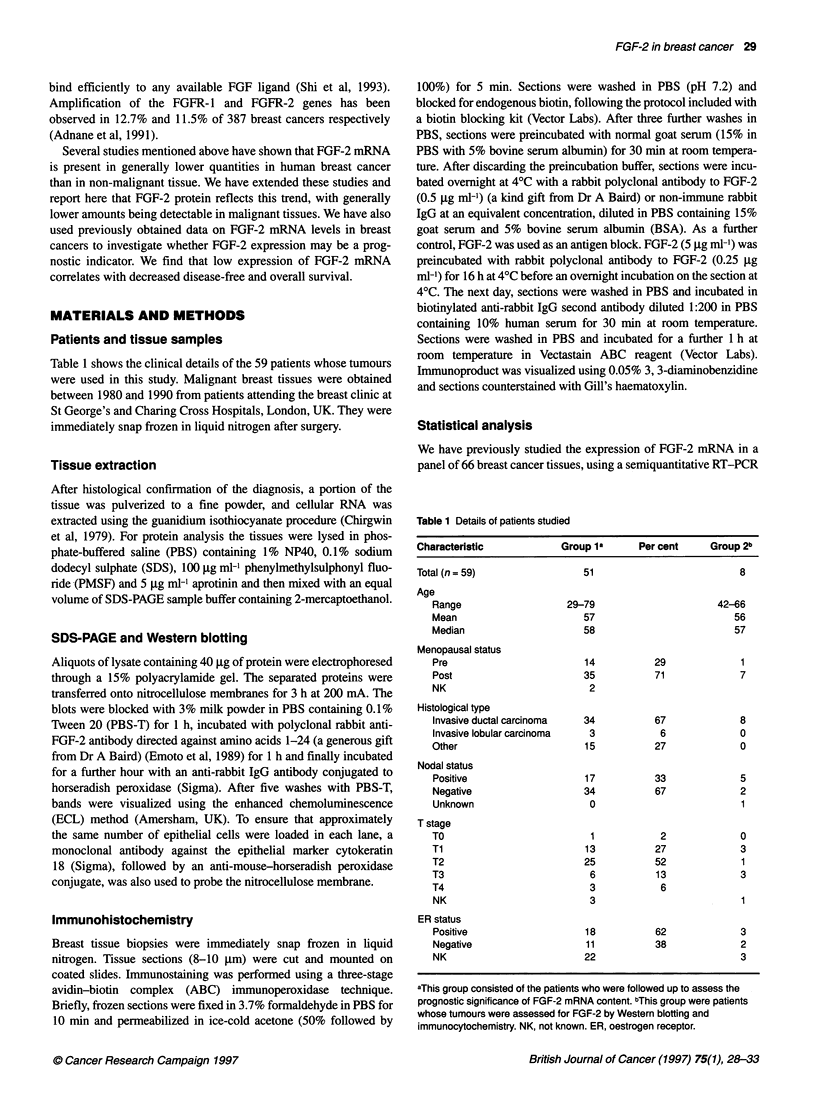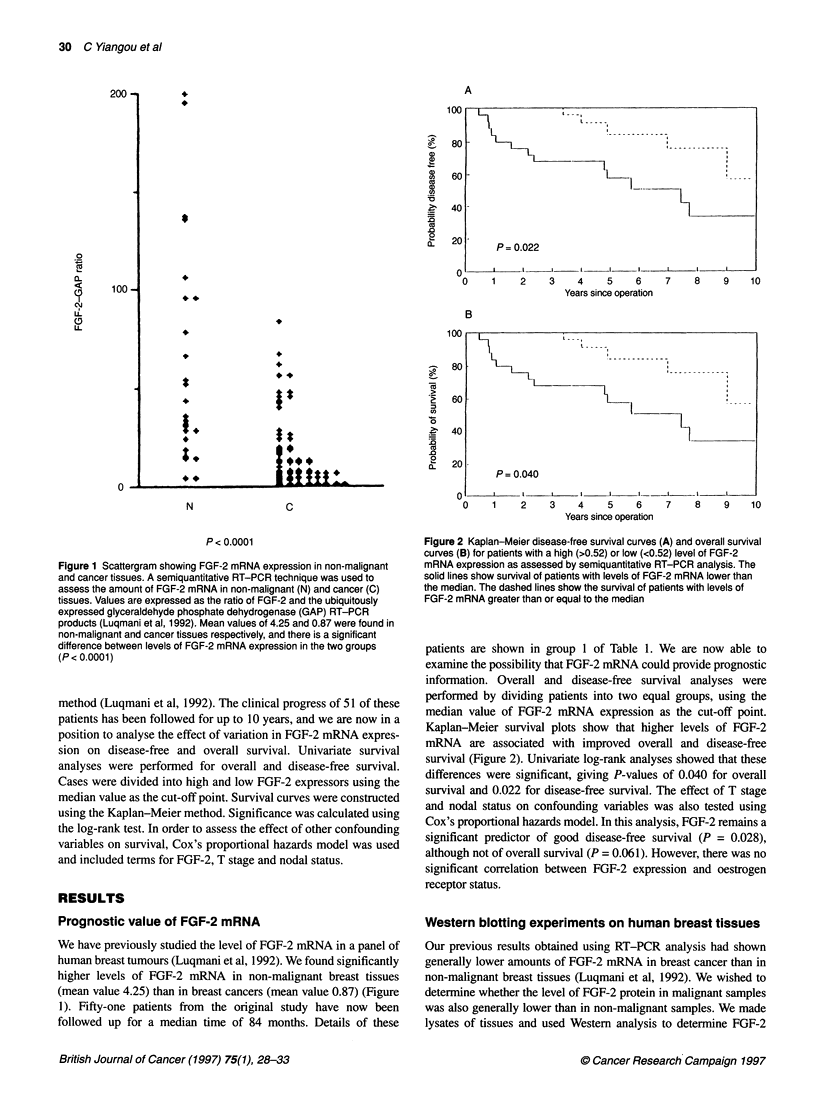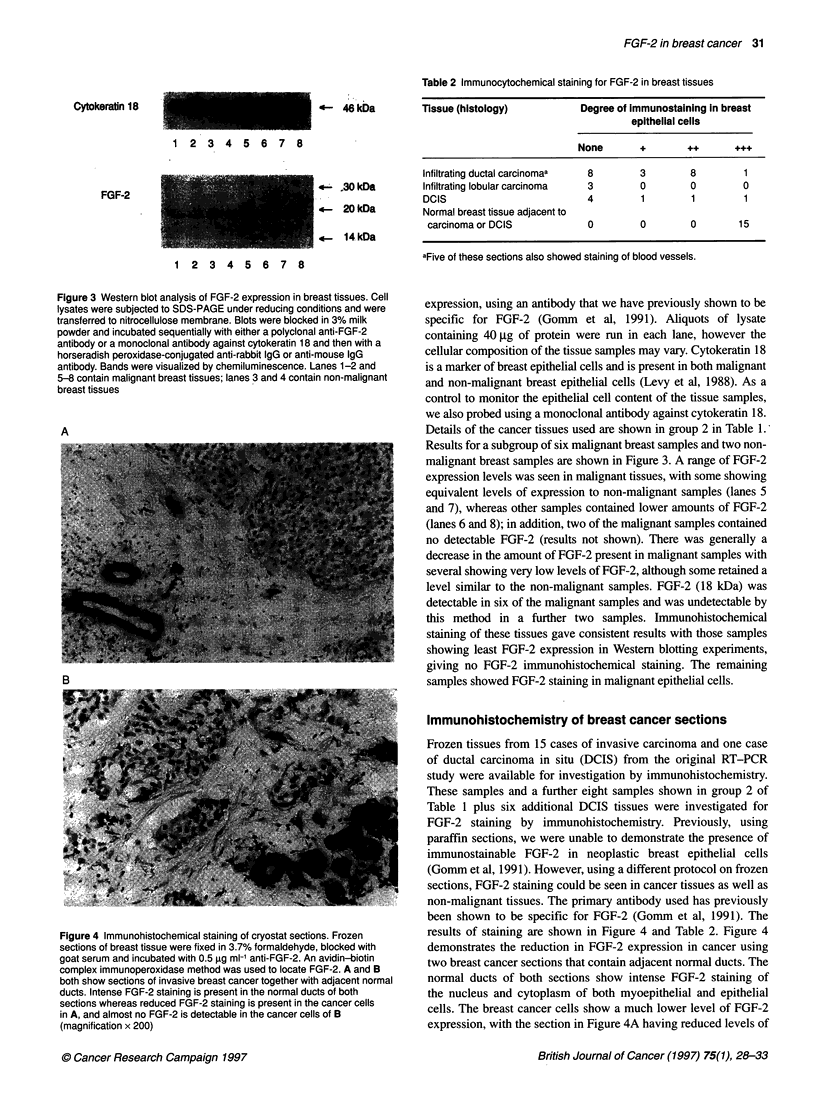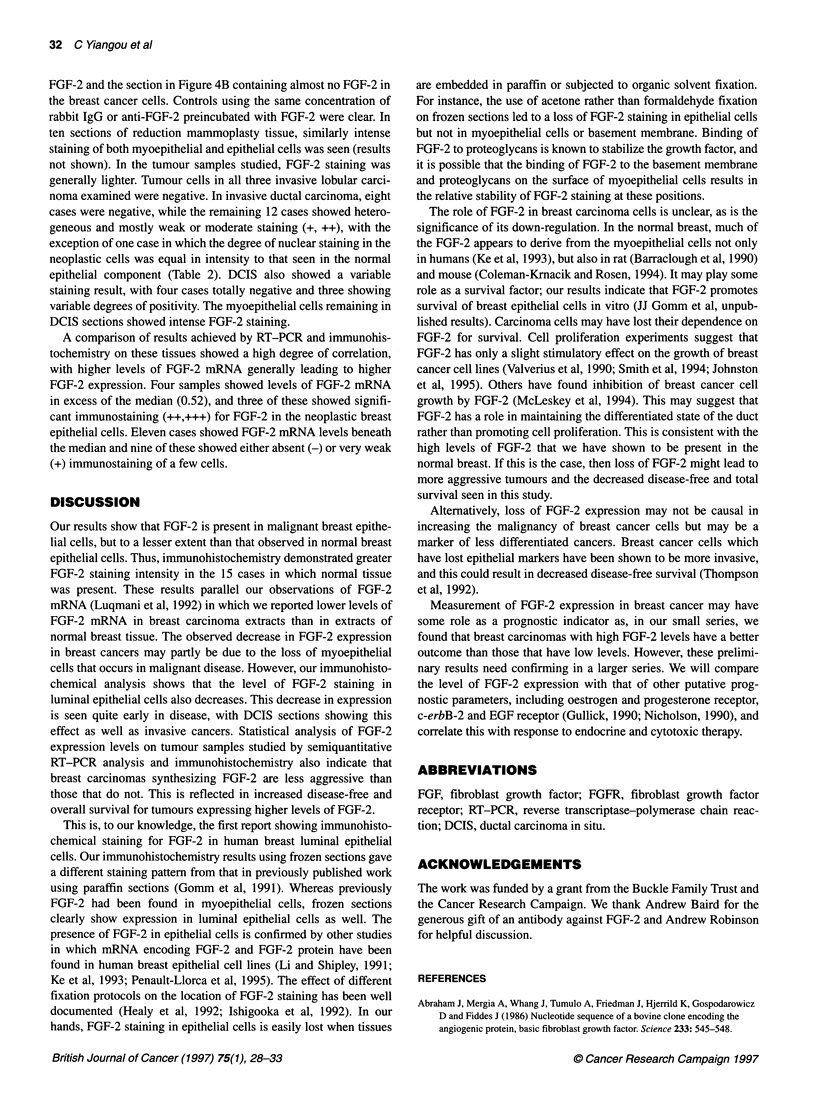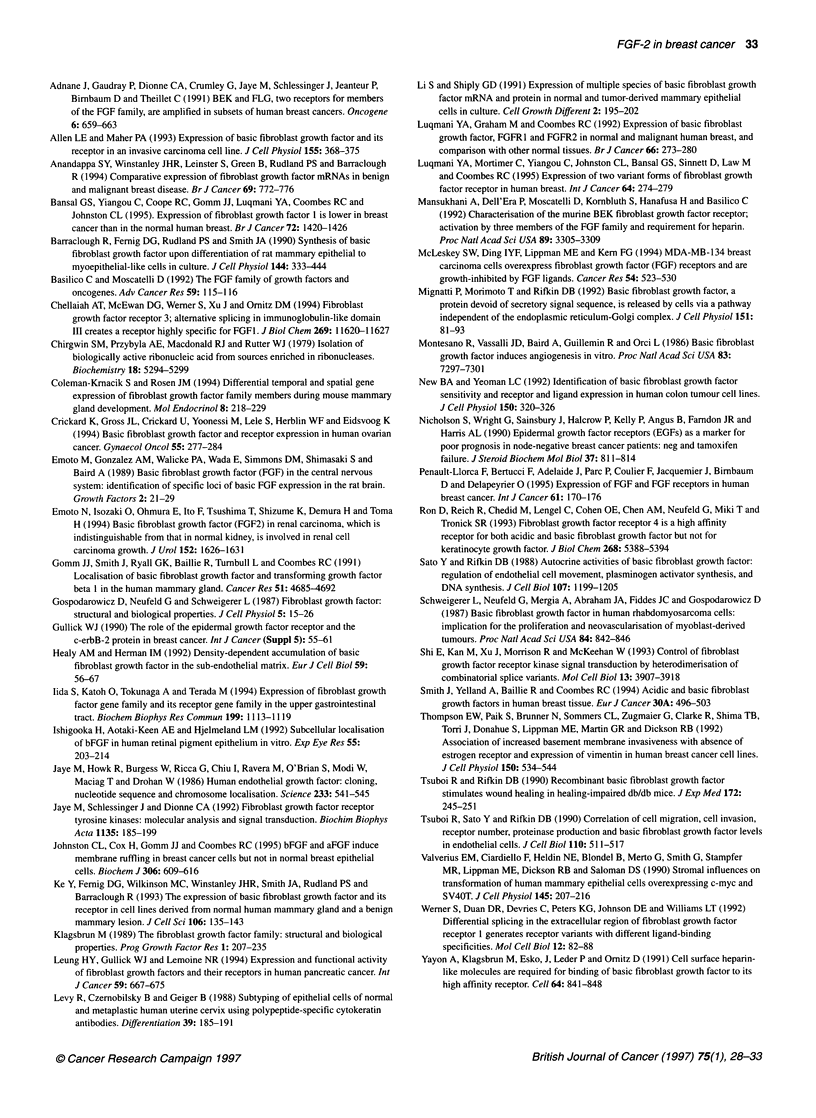# Fibroblast growth factor 2 in breast cancer: occurrence and prognostic significance.

**DOI:** 10.1038/bjc.1997.5

**Published:** 1997

**Authors:** C. Yiangou, J. J. Gomm, R. C. Coope, M. Law, Y. A. Luqmani, S. Shousha, R. C. Coombes, C. L. Johnston

**Affiliations:** Department of Medical Oncology, Charing Cross Hospital, London, UK.

## Abstract

**Images:**


British Journal of Cancer (1997) 75(1), 28–33
© 1997 Cancer Research Campaign

### Fibroblast growth factor 2 in breast cancer: occurrence and prognostic significance

C Yiangou[1], JJ Gomm[1], RC Coope[1], M Law[2], YA Luqmani[1,*], S Shousha[3], RC Coombes[1] and CL Johnston[1]

[1]Department of Medical Oncology, Charing Cross Hospital, London W6 8RF, UK; [2]Institute of Cancer Research, Royal Marsden Hospital, Surrey SM2 5NG, UK; [3]Department of Histopathology, Charing Cross Hospital, London W6 8RF, UK

**Summary** This paper examines the expression of fibroblast growth factor 2 (FGF-2) in the malignant human breast. Semiquantitative reverse transcriptase–polymerase chain reaction (RT–PCR) was used to assess the level of expression of FGF-2 in a series of 51 patients clinically followed up for a median of 84 months (Luqmani et al, 1992). Immunohistochemistry and Western blotting were used to show that the level of FGF-2 in breast tissues correlated with the amount of FGF-2 mRNA. FGF-2 was present in both malignant and non-malignant breast, although less was expressed in malignant tissues as determined by all three methods. Immunohistochemistry on frozen sections of breast tissue showed expression of FGF-2 in myoepithelial and epithelial cells in non-malignant samples and generally lower or undetectable levels of staining in malignant epithelial cells. The results obtained by immunohistochemistry correlated well with RT–PCR data showing similar levels of FGF-2 and FGF-2 mRNA expression in samples. No correlation was found between FGF-2 mRNA expression and T stage, nodal status or oestrogen receptor status. However, Kaplan–Meier survival plots show that higher levels of FGF-2 are associated with improved overall and disease-free survival. We suggest that FGF-2 expression may have value as a prognostic indicator in breast cancer.

**Keywords:** fibroblast growth factor; breast cancer; clinical prognosis

The fibroblast growth factors (FGFs) form a family of nine identified growth-regulatory proteins that share 35–50% overall homology and induce proliferation and differentiation of a wide range of cells of epithelial, mesodermal and neuroectodermal origin (Gospodarowicz et al, 1987; Basilico and Moscatelli, 1992; Klagsbrun, 1989). All except FGF-1 and FGF-2 are synthesized with an N-terminal hydrophobic signal sequence, enabling the classical mechanism of secretion from cells (Abraham et al, 1986; Jaye et al, 1986). Release of FGF-1 and FGF-2 may occur through leakage from damaged cells or from viable cells through a novel mechanism (Mignatti et al, 1992). FGF-2 has diverse biological functions, which include involvement in cellular growth, differentiation, embryogenesis, wound healing, plasminogen activator synthesis, invasion and angiogenesis (Montesano et al, 1986; Gospodarowicz et al, 1987; Sato and Rifkin, 1988; Klagsbrun, 1989; Tsuboi and Rifkin, 1990; Tsuboi et al, 1990). Several of these functions could influence the progression of cancer by encouraging cell growth or metastasis, and FGFs have consequently been studied in several cancers.

FGF-2 has been detected in a variety of human cancers, including colonic adenocarcinoma (New and Yeoman, 1992), bladder cancer (Allen and Maher, 1993), rhabdomyosarcoma (Schweigerer et al, 1987), ovarian cancer (Crickard et al, 1994), pancreatic carcinoma (Leung et al, 1994), renal cell carcinoma (Emoto et al, 1994) and oesophageal carcinoma (Iida et al, 1994). Studies of human breast have shown high levels of FGF-2 mRNA in non-malignant breast, with malignant transformation of epithelial cells leading to reduced expression (Luqmani et al, 1992; Anandappa et al, 1994). However, both studies showed that about one in four cancers expressed FGF-2 mRNA at levels similar to those found in the benign tissue. A similar pattern is seen for FGF-1 expression in the breast, as lower amounts of FGF-1 and FGF-1 mRNA were found in malignant breast tissue than in non-malignant breast tissue (Bansal et al, 1995). The principal source of FGF-2 in the breast appears to be myoepithelial cells. FGF-2 mRNA has been detected in both epithelial and myoepithelial breast cells, with the latter expressing higher levels (Ke et al, 1993). Immunohistochemical studies using paraffin sections have shown that FGF-2 localizes to myoepithelial cells and the basement membrane in benign breast biopsies and intraduct carcinomas (Gomm et al, 1991).

The cellular response to FGF-2 is mediated through the formation of a trimolecular complex composed of FGF-2, one of the high-affinity FGF receptors (FGFR) and an extracellular matrix or cell-surface heparan sulphate proteoglycan (Yayon et al, 1991). A family of four high-affinity receptors encoded by separate genes has been identified, and the complexity of this family is enhanced by an array of spliced variants (Jaye et al, 1992). The principal high-affinity receptors for FGF-2 appear to be FGFR-1 and one of the splice variants of FGFR-2 (FGFR-2-IIIC) (Mansukhani et al, 1992; Werner et al, 1992). FGFR-3 and FGFR-4 bind to FGF-2 with lower affinity (Ron et al, 1993; Chellaiah et al, 1994). Both FGFR-1 and the IIIc variant of FGFR-2 are present in breast cancers (Luqmani et al, 1992; Penault-Llorca et al, 1995). The relative level of the beta (2-immunoglobulin-like domain) form compared with the alpha (3-immunoglobulin-like domain) form of FGFR-1 is higher in malignant breast biopsies (Luqmani et al, 1995). The beta form of FGFR-1 has been reported to have a higher affinity for FGF-2, indicating that breast cancer cells may

*Received 4 March 1996*
*Revised 15 July 1996*
*Accepted 23 July 1996*

*Correspondence to:* CL Johnston

*Present address: Faculty of Allied Health Sciences, Kuwait University, PO Box 31470, Kuwait bind efficiently to any available FGF ligand (Shi et al, 1993). Amplification of the FGFR-1 and FGFR-2 genes has been observed in 12.7% and 11.5% of 387 breast cancers respectively (Adnane et al, 1991).

Several studies mentioned above have shown that FGF-2 mRNA is present in generally lower quantities in human breast cancer than in non-malignant tissue. We have extended these studies and report here that FGF-2 protein reflects this trend, with generally lower amounts being detectable in malignant tissues. We have also used previously obtained data on FGF-2 mRNA levels in breast cancers to investigate whether FGF-2 expression may be a prognostic indicator. We find that low expression of FGF-2 mRNA correlates with decreased disease-free and overall survival.

### MATERIALS AND METHODS

### Patients and tissue samples

Table 1 shows the clinical details of the 59 patients whose tumours were used in this study. Malignant breast tissues were obtained between 1980 and 1990 from patients attending the breast clinic at St George's and Charing Cross Hospitals, London, UK. They were immediately snap frozen in liquid nitrogen after surgery.

### Tissue extraction

After histological confirmation of the diagnosis, a portion of the tissue was pulverized to a fine powder, and cellular RNA was extracted using the guanidium isothiocyanate procedure (Chirgwin et al, 1979). For protein analysis the tissues were lysed in phosphate-buffered saline (PBS) containing 1% NP40, 0.1% sodium dodecyl sulphate (SDS), 100 μg $ml^{-1}$ phenylmethylsulphonyl fluoride (PMSF) and 5 μg $ml^{-1}$ aprotinin and then mixed with an equal volume of SDS-PAGE sample buffer containing 2-mercaptoethanol.

### SDS-PAGE and Western blotting

Aliquots of lysate containing 40 μg of protein were electrophoresed through a 15% polyacrylamide gel. The separated proteins were transferred onto nitrocellulose membranes for 3 h at 200 mA. The blots were blocked with 3% milk powder in PBS containing 0.1% Tween 20 (PBS-T) for 1 h, incubated with polyclonal rabbit anti-FGF-2 antibody directed against amino acids 1–24 (a generous gift from Dr A Baird) (Emoto et al, 1989) for 1 h and finally incubated for a further hour with an anti-rabbit IgG antibody conjugated to horseradish peroxidase (Sigma). After five washes with PBS-T, bands were visualized using the enhanced chemoluminescence (ECL) method (Amersham, UK). To ensure that approximately the same number of epithelial cells were loaded in each lane, a monoclonal antibody against the epithelial marker cytokeratin 18 (Sigma), followed by an anti-mouse–horseradish peroxidase conjugate, was also used to probe the nitrocellulose membrane.

### Immunohistochemistry

Breast tissue biopsies were immediately snap frozen in liquid nitrogen. Tissue sections (8–10 μm) were cut and mounted on coated slides. Immunostaining was performed using a three-stage avidin–biotin complex (ABC) immunoperoxidase technique. Briefly, frozen sections were fixed in 3.7% formaldehyde in PBS for 10 min and permeabilized in ice-cold acetone (50% followed by 100%) for 5 min. Sections were washed in PBS (pH 7.2) and blocked for endogenous biotin, following the protocol included with a biotin blocking kit (Vector Labs). After three further washes in PBS, sections were preincubated with normal goat serum (15% in PBS with 5% bovine serum albumin) for 30 min at room temperature. After discarding the preincubation buffer, sections were incubated overnight at 4°C with a rabbit polyclonal antibody to FGF-2 (0.5 μg $ml^{-1}$) (a kind gift from Dr A Baird) or non-immune rabbit IgG at an equivalent concentration, diluted in PBS containing 15% goat serum and 5% bovine serum albumin (BSA). As a further control, FGF-2 was used as an antigen block. FGF-2 (5 μg $ml^{-1}$) was preincubated with rabbit polyclonal antibody to FGF-2 (0.25 μg $ml^{-1}$) for 16 h at 4°C before an overnight incubation on the section at 4°C. The next day, sections were washed in PBS and incubated in biotinylated anti-rabbit IgG second antibody diluted 1:200 in PBS containing 10% human serum for 30 min at room temperature. Sections were washed in PBS and incubated for a further 1 h at room temperature in Vectastain ABC reagent (Vector Labs). Immunoproduct was visualized using 0.05% 3, 3-diaminobenzidine and sections counterstained with Gill's haematoxylin.

### Statistical analysis

We have previously studied the expression of FGF-2 mRNA in a panel of 66 breast cancer tissues, using a semiquantitative RT–PCR

**Table 1** Details of patients studied

| Characteristic | Group 1[a] | Per cent | Group 2[b] |
|---|---|---|---|
| Total ($n$ = 59) | 51 | | 8 |
| Age | | | |
| Range | 29–79 | | 42–66 |
| Mean | 57 | | 56 |
| Median | 58 | | 57 |
| Menopausal status | | | |
| Pre | 14 | 29 | 1 |
| Post | 35 | 71 | 7 |
| NK | 2 | | |
| Histological type | | | |
| Invasive ductal carcinoma | 34 | 67 | 8 |
| Invasive lobular carcinoma | 3 | 6 | 0 |
| Other | 15 | 27 | 0 |
| Nodal status | | | |
| Positive | 17 | 33 | 5 |
| Negative | 34 | 67 | 2 |
| Unknown | 0 | | 1 |
| T stage | | | |
| T0 | 1 | 2 | 0 |
| T1 | 13 | 27 | 3 |
| T2 | 25 | 52 | 1 |
| T3 | 6 | 13 | 3 |
| T4 | 3 | 6 | |
| NK | 3 | | 1 |
| ER status | | | |
| Positive | 18 | 62 | 3 |
| Negative | 11 | 38 | 2 |
| NK | 22 | | 3 |

[a]This group consisted of the patients who were followed up to assess the prognostic significance of FGF-2 mRNA content. [b]This group were patients whose tumours were assessed for FGF-2 by Western blotting and immunocytochemistry. NK, not known. ER, oestrogen receptor.

**Figure 1** Scattergram showing FGF-2 mRNA expression in non-malignant and cancer tissues. A semiquantitative RT–PCR technique was used to assess the amount of FGF-2 mRNA in non-malignant (N) and cancer (C) tissues. Values are expressed as the ratio of FGF-2 and the ubiquitously expressed glyceraldehyde phosphate dehydrogenase (GAP) RT–PCR products (Luqmani et al, 1992). Mean values of 4.25 and 0.87 were found in non-malignant and cancer tissues respectively, and there is a significant difference between levels of FGF-2 mRNA expression in the two groups ($P < 0.0001$)

**Figure 2** Kaplan–Meier disease-free survival curves (**A**) and overall survival curves (**B**) for patients with a high (>0.52) or low (<0.52) level of FGF-2 mRNA expression as assessed by semiquantitative RT–PCR analysis. The solid lines show survival of patients with levels of FGF-2 mRNA lower than the median. The dashed lines show the survival of patients with levels of FGF-2 mRNA greater than or equal to the median method (Luqmani et al, 1992). The clinical progress of 51 of these patients has been followed for up to 10 years, and we are now in a position to analyse the effect of variation in FGF-2 mRNA expression on disease-free and overall survival. Univariate survival analyses were performed for overall and disease-free survival. Cases were divided into high and low FGF-2 expressors using the median value as the cut-off point. Survival curves were constructed using the Kaplan–Meier method. Significance was calculated using the log-rank test. In order to assess the effect of other confounding variables on survival, Cox's proportional hazards model was used and included terms for FGF-2, T stage and nodal status.

### RESULTS

### Prognostic value of FGF-2 mRNA

We have previously studied the level of FGF-2 mRNA in a panel of human breast tumours (Luqmani et al, 1992). We found significantly higher levels of FGF-2 mRNA in non-malignant breast tissues (mean value 4.25) than in breast cancers (mean value 0.87) (Figure 1). Fifty-one patients from the original study have now been followed up for a median time of 84 months. Details of these patients are shown in group 1 of Table 1. We are now able to examine the possibility that FGF-2 mRNA could provide prognostic information. Overall and disease-free survival analyses were performed by dividing patients into two equal groups, using the median value of FGF-2 mRNA expression as the cut-off point. Kaplan–Meier survival plots show that higher levels of FGF-2 mRNA are associated with improved overall and disease-free survival (Figure 2). Univariate log-rank analyses showed that these differences were significant, giving *P*-values of 0.040 for overall survival and 0.022 for disease-free survival. The effect of T stage and nodal status on confounding variables was also tested using Cox's proportional hazards model. In this analysis, FGF-2 remains a significant predictor of good disease-free survival ($P = 0.028$), although not of overall survival ($P = 0.061$). However, there was no significant correlation between FGF-2 expression and oestrogen receptor status.

### Western blotting experiments on human breast tissues

Our previous results obtained using RT–PCR analysis had shown generally lower amounts of FGF-2 mRNA in breast cancer than in non-malignant breast tissues (Luqmani et al, 1992). We wished to determine whether the level of FGF-2 protein in malignant samples was also generally lower than in non-malignant samples. We made lysates of tissues and used Western analysis to determine FGF-2

© Cancer Research Campaign 1997

**Figure 3** Western blot analysis of FGF-2 expression in breast tissues. Cell lysates were subjected to SDS-PAGE under reducing conditions and were transferred to nitrocellulose membrane. Blots were blocked in 3% milk powder and incubated sequentially with either a polyclonal anti-FGF-2 antibody or a monoclonal antibody against cytokeratin 18 and then with a horseradish peroxidase-conjugated anti-rabbit IgG or anti-mouse IgG antibody. Bands were visualized by chemiluminescence. Lanes 1–2 and 5–8 contain malignant breast tissues; lanes 3 and 4 contain non-malignant breast tissues

**Figure 4** Immunohistochemical staining of cryostat sections. Frozen sections of breast tissue were fixed in 3.7% formaldehyde, blocked with goat serum and incubated with 0.5 μg ml$^{-1}$ anti-FGF-2. An avidin–biotin complex immunoperoxidase method was used to locate FGF-2. **A** and **B** both show sections of invasive breast cancer together with adjacent normal ducts. Intense FGF-2 staining is present in the normal ducts of both sections whereas reduced FGF-2 staining is present in the cancer cells in **A**, and almost no FGF-2 is detectable in the cancer cells of **B** (magnification × 200)

**Table 2** Immunocytochemical staining for FGF-2 in breast tissues

| Tissue (histology) | Degree of immunostaining in breast epithelial cells | | | |
|---|---|---|---|---|
| | None | + | ++ | +++ |
| Infiltrating ductal carcinoma[a] | 8 | 3 | 8 | 1 |
| Infiltrating lobular carcinoma | 3 | 0 | 0 | 0 |
| DCIS | 4 | 1 | 1 | 1 |
| Normal breast tissue adjacent to carcinoma or DCIS | 0 | 0 | 0 | 15 |

[a]Five of these sections also showed staining of blood vessels.

expression, using an antibody that we have previously shown to be specific for FGF-2 (Gomm et al, 1991). Aliquots of lysate containing 40 μg of protein were run in each lane, however the cellular composition of the tissue samples may vary. Cytokeratin 18 is a marker of breast epithelial cells and is present in both malignant and non-malignant breast epithelial cells (Levy et al, 1988). As a control to monitor the epithelial cell content of the tissue samples, we also probed using a monoclonal antibody against cytokeratin 18. Details of the cancer tissues used are shown in group 2 in Table 1. Results for a subgroup of six malignant breast samples and two non-malignant breast samples are shown in Figure 3. A range of FGF-2 expression levels was seen in malignant tissues, with some showing equivalent levels of expression to non-malignant samples (lanes 5 and 7), whereas other samples contained lower amounts of FGF-2 (lanes 6 and 8); in addition, two of the malignant samples contained no detectable FGF-2 (results not shown). There was generally a decrease in the amount of FGF-2 present in malignant samples with several showing very low levels of FGF-2, although some retained a level similar to the non-malignant samples. FGF-2 (18 kDa) was detectable in six of the malignant samples and was undetectable by this method in a further two samples. Immunohistochemical staining of these tissues gave consistent results with those samples showing least FGF-2 expression in Western blotting experiments, giving no FGF-2 immunohistochemical staining. The remaining samples showed FGF-2 staining in malignant epithelial cells.

### Immunohistochemistry of breast cancer sections

Frozen tissues from 15 cases of invasive carcinoma and one case of ductal carcinoma in situ (DCIS) from the original RT–PCR study were available for investigation by immunohistochemistry. These samples and a further eight samples shown in group 2 of Table 1 plus six additional DCIS tissues were investigated for FGF-2 staining by immunohistochemistry. Previously, using paraffin sections, we were unable to demonstrate the presence of immunostainable FGF-2 in neoplastic breast epithelial cells (Gomm et al, 1991). However, using a different protocol on frozen sections, FGF-2 staining could be seen in cancer tissues as well as non-malignant tissues. The primary antibody used has previously been shown to be specific for FGF-2 (Gomm et al, 1991). The results of staining are shown in Figure 4 and Table 2. Figure 4 demonstrates the reduction in FGF-2 expression in cancer using two breast cancer sections that contain adjacent normal ducts. The normal ducts of both sections show intense FGF-2 staining of the nucleus and cytoplasm of both myoepithelial and epithelial cells. The breast cancer cells show a much lower level of FGF-2 expression, with the section in Figure 4A having reduced levels of

© Cancer Research Campaign 1997

FGF-2 and the section in Figure 4B containing almost no FGF-2 in the breast cancer cells. Controls using the same concentration of rabbit IgG or anti-FGF-2 preincubated with FGF-2 were clear. In ten sections of reduction mammoplasty tissue, similarly intense staining of both myoepithelial and epithelial cells was seen (results not shown). In the tumour samples studied, FGF-2 staining was generally lighter. Tumour cells in all three invasive lobular carcinoma examined were negative. In invasive ductal carcinoma, eight cases were negative, while the remaining 12 cases showed heterogeneous and mostly weak or moderate staining (+, ++), with the exception of one case in which the degree of nuclear staining in the neoplastic cells was equal in intensity to that seen in the normal epithelial component (Table 2). DCIS also showed a variable staining result, with four cases totally negative and three showing variable degrees of positivity. The myoepithelial cells remaining in DCIS sections showed intense FGF-2 staining.

A comparison of results achieved by RT–PCR and immunohistochemistry on these tissues showed a high degree of correlation, with higher levels of FGF-2 mRNA generally leading to higher FGF-2 expression. Four samples showed levels of FGF-2 mRNA in excess of the median (0.52), and three of these showed significant immunostaining (++,+++) for FGF-2 in the neoplastic breast epithelial cells. Eleven cases showed FGF-2 mRNA levels beneath the median and nine of these showed either absent (–) or very weak (+) immunostaining of a few cells.

### DISCUSSION

Our results show that FGF-2 is present in malignant breast epithelial cells, but to a lesser extent than that observed in normal breast epithelial cells. Thus, immunohistochemistry demonstrated greater FGF-2 staining intensity in the 15 cases in which normal tissue was present. These results parallel our observations of FGF-2 mRNA (Luqmani et al, 1992) in which we reported lower levels of FGF-2 mRNA in breast carcinoma extracts than in extracts of normal breast tissue. The observed decrease in FGF-2 expression in breast cancers may partly be due to the loss of myoepithelial cells that occurs in malignant disease. However, our immunohistochemical analysis shows that the level of FGF-2 staining in luminal epithelial cells also decreases. This decrease in expression is seen quite early in disease, with DCIS sections showing this effect as well as invasive cancers. Statistical analysis of FGF-2 expression levels on tumour samples studied by semiquantitative RT–PCR analysis and immunohistochemistry also indicate that breast carcinomas synthesizing FGF-2 are less aggressive than those that do not. This is reflected in increased disease-free and overall survival for tumours expressing higher levels of FGF-2.

This is, to our knowledge, the first report showing immunohistochemical staining for FGF-2 in human breast luminal epithelial cells. Our immunohistochemistry results using frozen sections gave a different staining pattern from that in previously published work using paraffin sections (Gomm et al, 1991). Whereas previously FGF-2 had been found in myoepithelial cells, frozen sections clearly show expression in luminal epithelial cells as well. The presence of FGF-2 in epithelial cells is confirmed by other studies in which mRNA encoding FGF-2 and FGF-2 protein have been found in human breast epithelial cell lines (Li and Shipley, 1991; Ke et al, 1993; Penault-Llorca et al, 1995). The effect of different fixation protocols on the location of FGF-2 staining has been well documented (Healy et al, 1992; Ishigooka et al, 1992). In our hands, FGF-2 staining in epithelial cells is easily lost when tissues are embedded in paraffin or subjected to organic solvent fixation. For instance, the use of acetone rather than formaldehyde fixation on frozen sections led to a loss of FGF-2 staining in epithelial cells but not in myoepithelial cells or basement membrane. Binding of FGF-2 to proteoglycans is known to stabilize the growth factor, and it is possible that the binding of FGF-2 to the basement membrane and proteoglycans on the surface of myoepithelial cells results in the relative stability of FGF-2 staining at these positions.

The role of FGF-2 in breast carcinoma cells is unclear, as is the significance of its down-regulation. In the normal breast, much of the FGF-2 appears to derive from the myoepithelial cells not only in humans (Ke et al, 1993), but also in rat (Barraclough et al, 1990) and mouse (Coleman-Krnacik and Rosen, 1994). It may play some role as a survival factor; our results indicate that FGF-2 promotes survival of breast epithelial cells in vitro (JJ Gomm et al, unpublished results). Carcinoma cells may have lost their dependence on FGF-2 for survival. Cell proliferation experiments suggest that FGF-2 has only a slight stimulatory effect on the growth of breast cancer cell lines (Valverius et al, 1990; Smith et al, 1994; Johnston et al, 1995). Others have found inhibition of breast cancer cell growth by FGF-2 (McLeskey et al, 1994). This may suggest that FGF-2 has a role in maintaining the differentiated state of the duct rather than promoting cell proliferation. This is consistent with the high levels of FGF-2 that we have shown to be present in the normal breast. If this is the case, then loss of FGF-2 might lead to more aggressive tumours and the decreased disease-free and total survival seen in this study.

Alternatively, loss of FGF-2 expression may not be causal in increasing the malignancy of breast cancer cells but may be a marker of less differentiated cancers. Breast cancer cells which have lost epithelial markers have been shown to be more invasive, and this could result in decreased disease-free survival (Thompson et al, 1992).

Measurement of FGF-2 expression in breast cancer may have some role as a prognostic indicator as, in our small series, we found that breast carcinomas with high FGF-2 levels have a better outcome than those that have low levels. However, these preliminary results need confirming in a larger series. We will compare the level of FGF-2 expression with that of other putative prognostic parameters, including oestrogen and progesterone receptor, c-*erb*B-2 and EGF receptor (Gullick, 1990; Nicholson, 1990), and correlate this with response to endocrine and cytotoxic therapy.

### ABBREVIATIONS

FGF, fibroblast growth factor; FGFR, fibroblast growth factor receptor; RT–PCR, reverse transcriptase–polymerase chain reaction; DCIS, ductal carcinoma in situ.

### ACKNOWLEDGEMENTS

The work was funded by a grant from the Buckle Family Trust and the Cancer Research Campaign. We thank Andrew Baird for the generous gift of an antibody against FGF-2 and Andrew Robinson for helpful discussion.

### REFERENCES

Abraham J, Mergia A, Whang J, Tumulo A, Friedman J, Hjerrild K, Gospodarowicz D and Fiddes J (1986) Nucleotide sequence of a bovine clone encoding the angiogenic protein, basic fibroblast growth factor. *Science* **233:** 545–548.

 © Cancer Research Campaign 1997

Adnane J, Gaudray P, Dionne CA, Crumley G, Jaye M, Schlessinger J, Jeanteur P, Birnbaum D and Theillet C (1991) BEK and FLG, two receptors for members of the FGF family, are amplified in subsets of human breast cancers. *Oncogene* **6:** 659–663

Allen LE and Maher PA (1993) Expression of basic fibroblast growth factor and its receptor in an invasive carcinoma cell line. *J Cell Physiol* **155:** 368–375

Anandappa SY, Winstanley JHR, Leinster S, Green B, Rudland PS and Barraclough R (1994) Comparative expression of fibroblast growth factor mRNAs in benign and malignant breast disease. *Br J Cancer* **69:** 772–776

Bansal GS, Yiangou C, Coope RC, Gomm JJ, Luqmani YA, Coombes RC and Johnston CL (1995). Expression of fibroblast growth factor 1 is lower in breast cancer than in the normal human breast. *Br J Cancer* **72:** 1420–1426

Barraclough R, Fernig DG, Rudland PS and Smith JA (1990) Synthesis of basic fibroblast growth factor upon differentiation of rat mammary epithelial to myoepithelial-like cells in culture. *J Cell Physiol* **144:** 333–444

Basilico C and Moscatelli D (1992) The FGF family of growth factors and oncogenes. *Adv Cancer Res* **59:** 115–116

Chellaiah AT, McEwan DG, Werner S, Xu J and Ornitz DM (1994) Fibroblast growth factor receptor 3; alternative splicing in immunoglobulin-like domain III creates a receptor highly specific for FGF1. *J Biol Chem* **269:** 11620–11627

Chirgwin SM, Przybyla AE, Macdonald RJ and Rutter WJ (1979) Isolation of biologically active ribonucleic acid from sources enriched in ribonucleases. *Biochemistry* **18:** 5294–5299

Coleman-Krnacik S and Rosen JM (1994) Differential temporal and spatial gene expression of fibroblast growth factor family members during mouse mammary gland development. *Mol Endocrinol* **8:** 218–229

Crickard K, Gross JL, Crickard U, Yoonessi M, Lele S, Herblin WF and Eidsvoog K (1994) Basic fibroblast growth factor and receptor expression in human ovarian cancer. *Gynaecol Oncol* **55:** 277–284

Emoto M, Gonzalez AM, Walicke PA, Wada E, Simmons DM, Shimasaki S and Baird A (1989) Basic fibroblast growth factor (FGF) in the central nervous system: identification of specific loci of basic FGF expression in the rat brain. *Growth Factors* **2:** 21–29

Emoto N, Isozaki O, Ohmura E, Ito F, Tsushima T, Shizume K, Demura H and Toma H (1994) Basic fibroblast growth factor (FGF2) in renal carcinoma, which is indistinguishable from that in normal kidney, is involved in renal cell carcinoma growth. *J Urol* **152:** 1626–1631

Gomm JJ, Smith J, Ryall GK, Baillie R, Turnbull L and Coombes RC (1991) Localisation of basic fibroblast growth factor and transforming growth factor beta 1 in the human mammary gland. *Cancer Res* **51:** 4685–4692

Gospodarowicz D, Neufeld G and Schweigerer L (1987) Fibroblast growth factor: structural and biological properties. *J Cell Physiol* **5:** 15–26

Gullick WJ (1990) The role of the epidermal growth factor receptor and the c-erbB-2 protein in breast cancer. *Int J Cancer* **(Suppl 5):** 55–61

Healy AM and Herman IM (1992) Density-dependent accumulation of basic fibroblast growth factor in the sub-endothelial matrix. *Eur J Cell Biol* **59:** 56–67

Iida S, Katoh O, Tokunaga A and Terada M (1994) Expression of fibroblast growth factor gene family and its receptor gene family in the upper gastrointestinal tract. *Biochem Biophys Res Commun* **199:** 1113–1119

Ishigooka H, Aotaki-Keen AE and Hjelmeland LM (1992) Subcellular localisation of bFGF in human retinal pigment epithelium in vitro. *Exp Eye Res* **55:** 203–214

Jaye M, Howk R, Burgess W, Ricca G, Chiu I, Ravera M, O'Brian S, Modi W, Maciag T and Drohan W (1986) Human endothelial growth factor: cloning, nucleotide sequence and chromosome localisation. *Science* **233:** 541–545

Jaye M, Schlessinger J and Dionne CA (1992) Fibroblast growth factor receptor tyrosine kinases: molecular analysis and signal transduction. *Biochim Biophys Acta* **1135:** 185–199

Johnston CL, Cox H, Gomm JJ and Coombes RC (1995) bFGF and aFGF induce membrane ruffling in breast cancer cells but not in normal breast epithelial cells. *Biochem J* **306:** 609–616

Ke Y, Fernig DG, Wilkinson MC, Winstanley JHR, Smith JA, Rudland PS and Barraclough R (1993) The expression of basic fibroblast growth factor and its receptor in cell lines derived from normal human mammary gland and a benign mammary lesion. *J Cell Sci* **106:** 135–143

Klagsbrun M (1989) The fibroblast growth factor family: structural and biological properties. *Prog Growth Factor Res* **1:** 207–235

Leung HY, Gullick WJ and Lemoine NR (1994) Expression and functional activity of fibroblast growth factors and their receptors in human pancreatic cancer. *Int J Cancer* **59:** 667–675

Levy R, Czernobilsky B and Geiger B (1988) Subtyping of epithelial cells of normal and metaplastic human uterine cervix using polypeptide-specific cytokeratin antibodies. *Differentiation* **39:** 185–191

Li S and Shiply GD (1991) Expression of multiple species of basic fibroblast growth factor mRNA and protein in normal and tumor-derived mammary epithelial cells in culture. *Cell Growth Different* **2:** 195–202

Luqmani YA, Graham M and Coombes RC (1992) Expression of basic fibroblast growth factor, FGFR1 and FGFR2 in normal and malignant human breast, and comparison with other normal tissues. *Br J Cancer* **66:** 273–280

Luqmani YA, Mortimer C, Yiangou C, Johnston CL, Bansal GS, Sinnett D, Law M and Coombes RC (1995) Expression of two variant forms of fibroblast growth factor receptor in human breast. *Int J Cancer* **64:** 274–279

Mansukhani A, Dell'Era P, Moscatelli D, Kornbluth S, Hanafusa H and Basilico C (1992) Characterisation of the murine BEK fibroblast growth factor receptor; activation by three members of the FGF family and requirement for heparin. *Proc Natl Acad Sci USA* **89:** 3305–3309

McLeskey SW, Ding IYF, Lippman ME and Kern FG (1994) MDA-MB-134 breast carcinoma cells overexpress fibroblast growth factor (FGF) receptors and are growth-inhibited by FGF ligands. *Cancer Res* **54:** 523–530

Mignatti P, Morimoto T and Rifkin DB (1992) Basic fibroblast growth factor, a protein devoid of secretory signal sequence, is released by cells via a pathway independent of the endoplasmic reticulum-Golgi complex. *J Cell Physiol* **151:** 81–93

Montesano R, Vassalli JD, Baird A, Guillemin R and Orci L (1986) Basic fibroblast growth factor induces angiogenesis in vitro. *Proc Natl Acad Sci USA* **83:** 7297–7301

New BA and Yeoman LC (1992) Identification of basic fibroblast growth factor sensitivity and receptor and ligand expression in human colon tumour cell lines. *J Cell Physiol* **150:** 320–326

Nicholson S, Wright G, Sainsbury J, Halcrow P, Kelly P, Angus B, Farndon JR and Harris AL (1990) Epidermal growth factor receptors (EGFs) as a marker for poor prognosis in node-negative breast cancer patients: neg and tamoxifen failure. *J Steroid Biochem Mol Biol* **37:** 811–814

Penault-Llorca F, Bertucci F, Adelaide J, Parc P, Coulier F, Jacquemier J, Birnbaum D and Delapeyrier O (1995) Expression of FGF and FGF receptors in human breast cancer. *Int J Cancer* **61:** 170–176

Ron D, Reich R, Chedid M, Lengel C, Cohen OE, Chen AM, Neufeld G, Miki T and Tronick SR (1993) Fibroblast growth factor receptor 4 is a high affinity receptor for both acidic and basic fibroblast growth factor but not for keratinocyte growth factor. *J Biol Chem* **268:** 5388–5394

Sato Y and Rifkin DB (1988) Autocrine activities of basic fibroblast growth factor: regulation of endothelial cell movement, plasminogen activator synthesis, and DNA synthesis. *J Cell Biol* **107:** 1199–1205

Schweigerer L, Neufeld G, Mergia A, Abraham JA, Fiddes JC and Gospodarowicz D (1987) Basic fibroblast growth factor in human rhabdomyosarcoma cells: implication for the proliferation and neovascularisation of myoblast-derived tumours. *Proc Natl Acad Sci USA* **84:** 842–846

Shi E, Kan M, Xu J, Morrison R and McKeehan W (1993) Control of fibroblast growth factor receptor kinase signal transduction by heterodimerisation of combinatorial splice variants. *Mol Cell Biol* **13:** 3907–3918

Smith J, Yelland A, Baillie R and Coombes RC (1994) Acidic and basic fibroblast growth factors in human breast tissue. *Eur J Cancer* **30A:** 496–503

Thompson EW, Paik S, Brunner N, Sommers CL, Zugmaier G, Clarke R, Shima TB, Torri J, Donahue S, Lippman ME, Martin GR and Dickson RB (1992) Association of increased basement membrane invasiveness with absence of estrogen receptor and expression of vimentin in human breast cancer cell lines. *J Cell Physiol* **150:** 534–544

Tsuboi R and Rifkin DB (1990) Recombinant basic fibroblast growth factor stimulates wound healing in healing-impaired db/db mice. *J Exp Med* **172:** 245–251

Tsuboi R, Sato Y and Rifkin DB (1990) Correlation of cell migration, cell invasion, receptor number, proteinase production and basic fibroblast growth factor levels in endothelial cells. *J Cell Biol* **110:** 511–517

Valverius EM, Ciardiello F, Heldin NE, Blondel B, Merto G, Smith G, Stampfer MR, Lippman ME, Dickson RB and Saloman DS (1990) Stromal influences on transformation of human mammary epithelial cells overexpressing c-myc and SV40T. *J Cell Physiol* **145:** 207–216

Werner S, Duan DR, Devries C, Peters KG, Johnson DE and Williams LT (1992) Differential splicing in the extracellular region of fibroblast growth factor receptor 1 generates receptor variants with different ligand-binding specificities. *Mol Cell Biol* **12:** 82–88

Yayon A, Klagsbrun M, Esko, J, Leder P and Ornitz D (1991) Cell surface heparin-like molecules are required for binding of basic fibroblast growth factor to its high affinity receptor. *Cell* **64:** 841–848

© Cancer Research Campaign 1997